# A Perceiver-Centered Approach for Representing and Annotating Prosodic Functions in Performed Music

**DOI:** 10.3389/fpsyg.2022.886570

**Published:** 2022-07-21

**Authors:** Daniel Bedoya, Lawrence Fyfe, Elaine Chew

**Affiliations:** ^1^STMS – CNRS, IRCAM, Sorbonne Université, Ministère de la Culture, Paris, France; ^2^Department of Engineering, King's College London, London, United Kingdom

**Keywords:** prosody, segmentation, prominence, annotation, representation, music performance

## Abstract

Musical prosody is characterized by the acoustic variations that make music expressive. However, few systematic and scalable studies exist on the function it serves or on effective tools to carry out such studies. To address this gap, we introduce a novel approach to capturing information about prosodic functions through a citizen science paradigm. In typical bottom-up approaches to studying musical prosody, acoustic properties in performed music and basic musical structures such as accents and phrases are mapped to prosodic functions, namely segmentation and prominence. In contrast, our top-down, human-centered method puts listener annotations of musical prosodic functions first, to analyze the connection between these functions, the underlying musical structures, and acoustic properties. The method is applied primarily to the exploring of segmentation and prominence in performed solo piano music. These prosodic functions are marked by means of four annotation types—boundaries, regions, note groups, and comments—in the CosmoNote web-based citizen science platform, which presents the music signal or MIDI data and related acoustic features in information layers that can be toggled on and off. Various annotation strategies are discussed and appraised: intuitive vs. analytical; real-time vs. retrospective; and, audio-based vs. visual. The end-to-end process of the data collection is described, from the providing of prosodic examples to the structuring and formatting of the annotation data for analysis, to techniques for preventing precision errors. The aim is to obtain reliable and coherent annotations that can be applied to theoretical and data-driven models of musical prosody. The outcomes include a growing library of prosodic examples with the goal of achieving an annotation convention for studying musical prosody in performed music.

## 1. Introduction

Acoustic variations in music can be studied through the concept of musical prosody. The term musical prosody is described by Palmer and Hutchins (2006) as the way acoustic properties are manipulated by performers to be expressive, without changing any existing categorical information (e.g., the pitch and duration categories of the notes in a score remain the same). Musical prosody can be applied to musical concepts such as melodic salience, expressive accents, musical phrases, and pauses. This view of music prosody borrows from speech, in particular, linguistics research that focuses on the phonetic features of speech sounds. In speech, prosody refers to phenomena involving the physical parameters of sound and the functions they serve in linguistic structure. The study of speech prosody is concerned, for example, with how frequency, duration, and intensity are used in stress, rhythm, and intonation (Speer and Blodgett, 2006), which has parallels in music. Music in and of itself can exhibit prosody, just like speech. Our focus here is on the prosody of the music content itself, although the term musical prosody has also been used to refer to that of the lyrics embedded in a song, which is often related to, but distinct from, that of the music content. For example, work by Migliore and Obin (2018) defines and analyzes musical prosody as a function of the “syllables of the words and beats of the measure”. In the remainder of the paper, we use the definition of musical prosody as given by Palmer and Hutchins (2006) and applied to instrumental music.

In Palmer and Hutchins (2006), musical prosody is described as carrying out four main functions: (1) segmentation (separating relevant elements in the music), (2) prominence (highlighting important events), (3) coordination (communicating with other performers while playing together), and (4) emotional response (reactions that the audience experiences when listening to the performance). We concentrate on the first two functions of musical prosody: segmentation and prominence. It should be pointed out that although segmentation and prominence are two distinct functions of musical prosody, they are not mutually exclusive. For instance, prominence is employed extensively by performers with the goal of highlighting segmentation, and by segmenting the music, changes in it are made more prominent. For the purpose of this paper, we present applications of our method to solo instrumental piano music, so we will not include studies of coordination. Emotional response is outside the scope of this paper, although our method could also be used to study and explain emotional reactions to music.

The definition of musical prosody as a topic is generally centered on studying how specific musical constructs, with or without considering their acoustic correlates, serve a given prosodic function. Palmer and Hutchins (2006) report how tempo, intensity and pitch modulations indicate the hierarchy of phrases, how articulations mark metrically important events, and how a tone duration can be mistaken when placed in an unexpected position in a phrase, to name just a few examples. This can be viewed as a bottom-up approach to musical prosody. Keeping the definition of musical prosody, we propose to explore its meanings in a different way, from a top-down perspective. That is, using the functions of segmentation and prominence as a starting point, given these constructs, we seek to understand how they are created. Ultimately, this will allow us to model the relationships between prosodic functions and the acoustic properties that form them, giving us a more complete understanding of these structures in performed music.

In developing our method, we asked two questions: (1) What kind of framework is necessary to represent and annotate segmentation and prominence? and, (2) how can we harness human perception to find these structures in performed music?

To answer the first question, we took inspiration from annotation protocols in speech, more specifically, from the ToBI (**To**nes and **B**reak **I**ndices) annotation standard. ToBI is a system that enables the transcription and annotation of speech prosody based on two concepts: tonal events (pitch accents, boundary tones, and phrase accents) and break indices (cues about phrase segmentation into words). Annotations are created in tiers and have their own labels. For example, the prosodic grouping of words is marked by vertical lines that have five different levels from 0 (most conjoint) to 4 (most disjoint) (Beckman and Ayers, 1997). Large communities of annotators have created their own versions of ToBI for multiple languages, each one with its own rules based on a specific language's prosodic structure. In a similar fashion, we have created the CosmoNote annotation platform (described in Section 2.2), drawing from the ToBI logic, for creating complex performed music structure visualizations and annotations.

To respond to the second question, we employ the citizen science paradigm to devise an experimental protocol aimed at gathering annotations on musical prosody. The umbrella term citizen science, is broadly defined across different fields encompassing many scientific goals. We defer to its characterization by Haklay et al. (2021) of participatory practices where people, called *citizen scientists*, get involved in research (e.g., collecting and analyzing data) without it being a part of their paid work. Some projects in citizen science encourage participants to adopt larger roles as they learn about the research subject, others foster co-creation of the research goals with members of the community to solve real-world problems (Senabre Hidalgo et al., 2021). This type of research is of interest because it allows researchers to reach a diverse population of people (who in our case enjoy music), and who have the potential to be involved in a project beyond the role of data gatherers, for example, by volunteering their thinking and reasoning capacities.

The rest of this article is organized as follows: Section 2 presents the CosmoNote web-based citizen science platform and other technical details of the protocol. Section 3 introduces the annotation method as applied to the prosodic functions of segmentation and prominence. While the specific musical structures that make up these functions are not fully known, we mention some plausible performed music structures within these categories, and recount some strategies for marking these structures systematically. Sections 4 and 5 discuss the anticipated results from collected data and the implications of answering our research questions for music performance science.

## 2. Materials

In order to study what are the structures that musicians create in performance, and how they could be represented, we need a set of tools to annotate listeners' and performers' perceptions of structures. Our approach is to record people's annotations of performed structures through a computer interface in intuitive and human-friendly ways as close as possible to how performers and listeners might think of these structures. The conveniences afforded by a digital platform that can be exploited include being able to see multiple layers of representations encoding different types of information, the ease of recognizing patterns over small and large time scales, and the ability to automate and scale certain annotation actions. Furthermore, annotations created in software have the advantage of being easily editable, accessible, and shareable.

There are several well-known computational tools for annotating, representing, and analyzing time-based media like music, speech, and video. In the realm of audio, many offer ways to markup structures in visual representations of audio descriptors such as the waveform, spectrogram, fundamental frequency, spectral centroid, or some similarity matrix. For example, Linguists use Praat (Boersma and Van Heuven, 2001) and ELAN (Brugman et al., 2004) to analyze, transcribe and annotate speech and video; musicologists use Sonic Visualizer (Cannam et al., 2010) and its vamp plugins, Eanalyse (Couprie, 2012), and Telemeta (Fillon et al., 2014) to study, analyze, and mark up electroacoustic music with or without collaborative web access.

### 2.1. Recordings and Data From Performances

In order to capture performed structures, we start with performance data, made up of audio and MIDI recordings along with the accompanying XML score of the music that was recorded. We record audio and MIDI and from live performances by professional pianists on a Bösendorfer 280VC ENSPIRE PRO Disklavier. Music representations and descriptors are extracted automatically from these source files.

We also use pre-recorded MIDI from various performance collections including the Bösendorfer Legendary Artists Library, or audio and MIDI from the Stanford Piano Roll Archive (SUPRA), and Steinway's Glenn Gould Goldberg Variations—MIDI files of the 1955 recording meticulously created for re-performances by Zenph Studios.

### 2.2. The CosmoNote Annotation Tool

**Figure 1:**
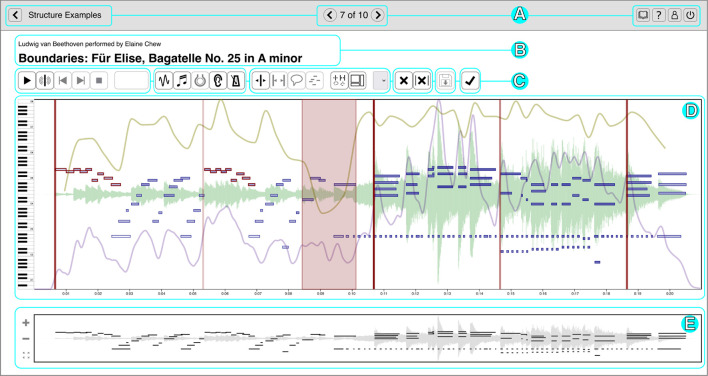
The main CosmoNote interface showing an annotated excerpt of Beethoven's Für Elise. The cyan rectangles highlight the different elements: **(A)** Navigation bar for the collection and account options; **(B)** Names of the piece, performer, composer; **(C)** Audio controls, annotation types, and other control buttons; **(D)** Visualization pane—annotations are displayed in shades of red on top of the waveform (pale green), piano roll (shades of blue), loudness (mauve), tempo (olive), and harmonic tension (shades of brown, not shown); and **(E)** Contextual zoom. See Supplementary Video 1 in the Supplementary Materials to hear the music presented in this Figure.

To present our performance data to participants, we load the data into CosmoNote^1^, a web-based interactive annotation platform that we built specifically for this purpose. It is custom software that facilitates music annotation tasks through an accessible interface that is both familiar for music enthusiasts and powerful enough for advanced users. CosmoNote was created to fill the gap in online collaborative markup tools by having both standard music representations as well as other descriptors important for expressiveness (Fyfe et al., 2021) such as loudness, tempo, and harmonic tension (see Figures 1A–E).

#### 2.2.1. Audio and Visualizations

Participants can listen to the performances as well as view various visualizations derived from the performance data. The waveform visualization provides a graphical representation of the music sound wave, giving a classic indication of the intensity and duration of sounds in the music. The piano roll visualization shows the notes or pitches over time along with their onset velocities (an approximation of loudness). The darkness of each note is an indicator of its velocity (darker is louder, lighter is quieter) and the length represents its duration. Sustain, soft, and sostenuto pedals are shown as graphical areas of different colors, showing the depth to which each pedal is pushed down; the color is a marker of the pedal type.

Additionally, three types of descriptors are computed from the audio, MIDI, and score data. Perceived loudness is estimated per frequency band using a psychoacoustic model implemented by Pampalk (2004) and plotted as a single smoothed, scaled curve. Tempo is computed using the inter-beat interval from automatic score alignment (Nakamura et al., 2017) or manual annotations when automatic alignment fails. Harmonic tension uses the spiral array model of tonality to visualize the dissonance (cloud diameter), chord change rate (cloud momentum), and distance from the tonal key (tensile strain) (Herremans and Chew, 2016).

#### 2.2.2. Annotations

Participants mark performance structures using four annotation types: boundaries of varying strengths (numbered 1 through 4), regions, note groups, and comments. Each of these constructs can be assigned their own labels and may be combined to denote a given performed structure. Annotations are displayed in shades of red on top of other visualization layers. Although allowing custom color coding for annotations could be useful, the visualization layers already use different colors. We thus decided to represent all annotations with red hues for a high visual contrast against other data. Annotations can be placed either by using mouse clicks or by using the keyboard. Embedded in each annotation type are several properties useful for analysis like the date of creation, timestamp (*start-end* for regions and just the *start* for all the rest), strength (for boundaries), and note information (note groups).

**Figure 2:**
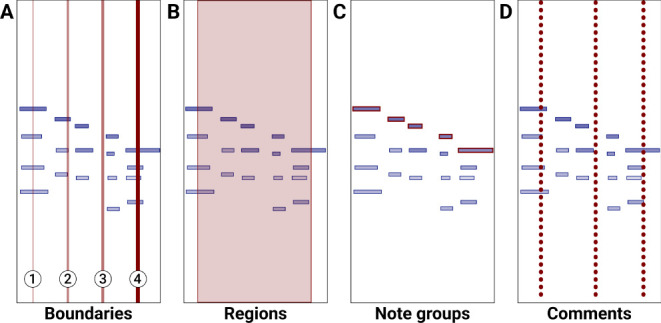
CosmoNote's four annotation types (shades of red) placed over the same sequence of notes (blue rectangles). **(A)** Four boundaries of different levels; **(B)** One region; **(C)** One note group containing five notes; **(D)** Three comments.

**Boundaries** were primarily designed to mark segmentation. They are drawn as vertical lines that span the whole visualization pane height. Boundaries have 4 increasing strength levels (from 1 to 4) and are displayed as a function of the line's thickness and its transparency (see Figure 2A). The choice of 4 boundary strengths was made to provide granularity without overwhelming the annotators with too many options. Boundaries can optionally be placed with the keyboard while the sound is playing using the numbers 1–4, allowing for placement while listening.

**Regions** are multi-functional annotation types that highlight all the elements inside a temporal selection with defined start and end times. Regions are drawn as semi-transparent red rectangles that span the whole visualization pane height (see Figure 2B). If two regions overlap, for example by marking the start of region *Y* before the end of region *X*, their individual transparencies are summed as a visual cue of the overlapping area.

**Note groups** are meant to single out individual salient notes, or groups of notes, that are meaningful in a given segment or prominent structure like a tipping point. Note groups are drawn as red rectangles on top of the normal blue rectangles that represent the notes of the piano roll visualization (see Figure 2C). Individual notes can be shared between different note groups.

**Comments** can be used as a way to write an observation about the performance at a specific time that is not otherwise represented by the other annotation types. They are drawn as dotted lines that span the whole height of the visualization pane (see Figure 2D).

### 2.3. Participants

Participants are citizen scientists interested in music and the research goals of discovering how performers create structures in performances and how listeners understand them. In order to use the interface to annotate the recorded performances, the user should have either headphones or speakers, and a computer with stable internet access. Participants first create an account using a valid email address and password to access the CosmoNote annotation platform, agreeing to the terms of the study and that they are over the age of 18.^2^ At a time of their choosing, participants may answer a short questionnaire (see Supplementary Materials). adapted from the Goldsmiths Musical Sophistication Index (Gold-MSI, by Müllensiefen et al., 2014) to describe their relationship to music in a more nuanced way than a simple *musician*-*non-musician* classification. Even though the annotation conventions are suitable for expert performers who may annotate their own work, no formal musical training is assumed or needed to contribute. An audio calibration stage allows participants to adjust their sound volume to a comfortable listening level and researchers to learn about the users' listening environments, i.e., their sound reproduction system and their hearing. It is based on a procedure by Cartwright et al. (2016) whereby participants are asked to count a number of random, equally loud, pure tones.

A training collection allowing participants to familiarize themselves with the interface and the annotations is always accessible to all annotators before completing the main annotation tasks. This training module currently features 3 short excerpts (around 20 s each) of the following pieces: Beethoven's “*Für Elise, Bagatelle No. 25 in A minor*”, Bach's “*Minuet in G minor BWV Anh 115*”, and Beethoven's “*Symphony No. 5 in C minor, Mvt II*”. The excerpts were chosen for being both simple and familiar to a wide audience while exhibiting good examples of prosodic functions in music. This collection is a sandbox environment where participants can create and save annotations that won't impact their work with the actual task. In addition, the main CosmoNote Youtube channel^3^ (also accessible from the CosmoNote website) has training material in the form of video examples showing how to place the different annotations.

### 2.4. Getting Feedback

After annotating a full set of performances in a collection, participants are invited to answer a questionnaire, giving feedback about their experience with the interface and with the annotation task. For example, for a boundary annotation task, the instructions will indicate the intent of boundary strengths while the feedback questionnaire will ask about the strategies annotators used, which will tell us how participants viewed/used the tools (see Supplementary Materials). As the experience and/or task may be different depending on the collection, custom feedback questionnaires can be shown for each collection. Participants are only asked to provide feedback after annotating a full collection because tasks usually involve annotating many pieces with similar properties. If a collection involved pieces without shared properties, a simplified version of the questionnaire could be implemented in the future. In this phase of studies, we want to minimize the time participants spend answering questionnaires and maximize the time they spend annotating music. Incidentally, annotators can include text labels on every annotation type and the special “Comment” annotation type is designed for immediate feedback (i.e., it allows users to mark something they find interesting or out of the ordinary). Although these questionnaires are the main avenue for receiving feedback, some studies will involve direct conversations with participants. Additionally, participants can reach the CosmoNote team *via* email (shown on the main site) for any other observations or comments they may have. The feedback will be used to iteratively refine the CosmoNote interface over subsequent annotation campaigns.

## 3. The Annotation Task

The performance annotation task, done with CosmoNote, is central to our method. Participants listen to audio recordings of the recorded piano performances while viewing the various music visualization layers (see Section 2.2 and Figure 2D) and are asked to mark segmentation and prominence in the music (as detailed in Sections 3.1 and 3.2). Participants are provided with annotations instructions that they can access at any time. Annotators currently have to read approximately one page of instructions though the exact amount of instruction/training needed may itself be the subject of future studies. Annotation instructions are tailored to specific collections of recorded performances, allowing us to run complementary studies as needed. There is no time constraint for making annotations. This means that participants will have the option of completing a set of annotations over multiple listening sessions, in a recurrent fashion. They may also revise their annotations over separate hearings before clicking the finish button which freezes the annotations, no longer allowing further changes.

**Figure 3:**
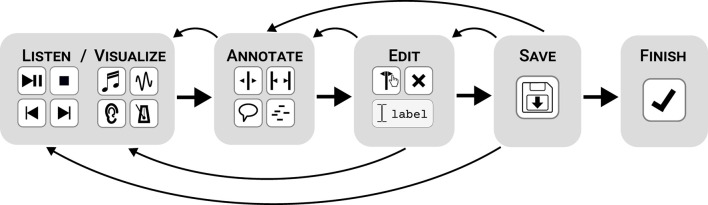
A diagram of 5 steps proposed to listeners for annotating a piece using CosmoNote, from left to right: listening/visualizing, annotating, editing, saving, and finishing. Notice that users are able to go back and forth between all intermediary stages until finishing.

Figure 3 shows an organic annotation workflow highlighting the five most frequent interface interactions that listeners are faced with when marking musical prosody in CosmoNote: (1) listening/visualizing (using the sound controls and visualization options), (2) annotating (placing any of the four annotation types), (3) editing (adding labels to, moving, or deleting annotations), (4) saving (syncing to the database), and (5) finishing (concluding the process for a given piece). The following subsections describe the annotating of segmentation and prominence respectively. It should be understood that these are not necessarily separate tasks but rather are two areas of focus within the overall annotation task.

### 3.1. Annotating Segmentation

Segmentation is the process of dividing something, in this case music, into meaningful units. It has been extensively studied in the fields of phonetics, speech perception, music analysis, and music information retrieval. For example, music theorists have famously studied the grouping processes that segment music into coherent chunks (Lerdahl and Jackendoff, 1996; Cambouropoulos, 2006), and how musical phrases, note lengthening, and intensity variations signal important segments to listeners.

Although prosodic cues can arise from composer-determined structures, our approach is focused on interpretive (that is performer-specific) use of musical prosody to segment musical streams. As a starting point, we examine how generalized concepts (Boundaries, Transitions, and Pauses) are traditionally defined, and in which ways they are likely to be shaped by performers.

**Figure 4:**
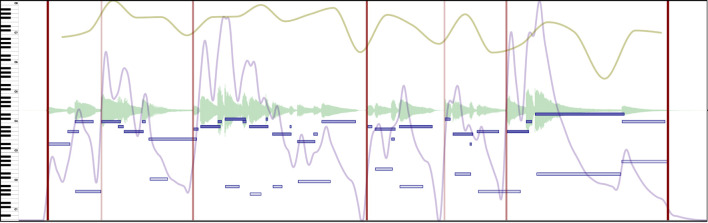
Excerpt of Beethoven's Symphony No. 5 in C minor, Mvt II. Boundaries (red vertical lines) in this example are assigned according to the strength of the change. Stronger boundaries have a thicker, less opaque red color. See Supplementary Video 2 in the Supplementary Materials to hear the music presented in this Figure.

**Boundaries** are well-studied structures that are used to describe segmentation (Wang et al., 2017). Even though their precise location can be ambiguous, annotated boundaries should indicate, by definition, clear points in time dividing the music stream into segments. These segments should be coherent (e.g., a complete musical idea or a musical thought) and help listeners make sense of the music. Figure 4 shows example boundary annotations.

Segmentation is mainly marked with the *Boundary* annotation type in CosmoNote. The four level *strengths* offer more granularity and provide more details for the data analysis. It is worth mentioning that, for this protocol, boundaries do not act as a nested hierarchy. This means for example that a boundary of level 1 is not contained in superior levels (2, 3, 4); and by extension, only one level is allowed at each time point. We can make sure that annotators understand how different boundary strengths mark segmentation (from 1-weak to 4-strong) by giving clear instructions. Boundary profiles aggregated from data of many participants will minimize effects of blunders (e.g., an annotator meant to press 1 but pressed 3) and outliers (e.g., an annotator marked a boundary where no one else did). Boundary levels can be a priori mapped to many types of segments. We describe below one of many possible mappings.

*Motives*, are the smallest indivisible succession of notes and/or rhythms detectable as a unit in music (Kennedy and Kennedy, 2013). They may be delineated by subtle cues like accents or micro pauses executed by the performer. In speech, letters attain meaning when they are turned into words. Likewise, individual notes or rhythms are imbued with meaning when grouped into musical motives. Musical motives are often repeated in a piece and may represent different concepts such as: the seed of a musical idea to be developed (e.g., the main motive in Beethoven's First Movement of his 5^th^ Symphony in C minor) or symbolize a character or an idea (e.g., in opera, a sequence of notes called *leitmotif* is repeated each time a character enters the scene). When motives have a larger priority in a piece, they are also called *figures*.

*Sub-phrases* are parts of a *Phrase*, which is a complete self-contained musical statement. Phrases and sub-phrases are often notated on the score using slurs, which give articulation cues to the performer. It is noticeable that these musical terms are directly linked to phrases in speech, which are governed by syntax. In that sense, the harmonic structure of a phrase (in tonal music) usually follows a set of syntactic rules (Rohrmeier and Pearce, 2018) that help to punctuate where musical phrases end; for example by using a cadence to arrive at a resting melodic or harmonic position. A common case of sub-phrases that constitute a larger phrase are antecedent and consequent sub-phrases, which resemble each other rhythmically and are complementary to each other.

*Sections* as defined by Spencer and Temko (1994) are major structural units made up of a number of smaller structural phenomena. They contain, for example, phrases and motives that are related between them and function as larger parts of a whole inside a piece. Sections represent a complete, but not independent musical idea, which is why pieces are generally composed of more than one section. Since they represent important parts of a piece, composers may notate sections in a score using double bar lines and they are often labeled alphabetically in musicological analysis with capital letters (e.g., *A, B, A'*). Similar to what happens with phrases, the end of a section is usually demarcated with some concluding melodic or harmonic device which is perceived as being conclusive; although the resolution is often clearer with sections. For example, performers may demarcate sections using a longer pause or larger tempo change compared to what they do with other boundaries.

**Figure 5:**
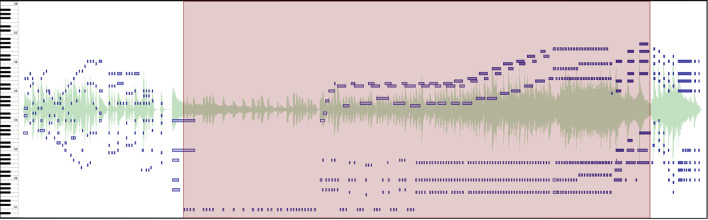
Excerpt of a long transition marked with a region (light red rectangle) between Movements III and IV of Beethoven's Symphony No. 5 in C minor. See Supplementary Video 3 in the Supplementary Materials to hear the music presented in this Figure.

**Transitions** may be musical passages that set up a change that is coming in the music, for example from one idea to the next. They can be seen as a link between sequential musical ideas. Transitions typically blur changes (boundaries) in the music by moving slowly through them. There are many ways to introduce a transition in music. For instance, elements from the following structure could be hinted at (e.g., a forthcoming motive is heard in a secondary voice) and/or new phrases can be introduced specifically to function as a bridge between ideas (e.g., the last passage of the third movement of Beethoven's 5^th^ symphony shown in Figure 5 transitions to the fourth movement). To execute a transition musically, performers can use tempo, dynamics and articulation to prepare the listener for what is coming by intentionally making the contrast between musical ideas smoother and less rigid.

**Figure 6:**
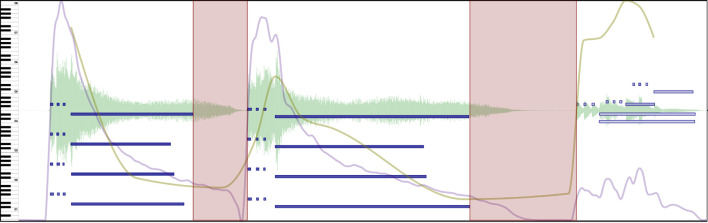
Two pauses marked with regions (light red rectangles) in an excerpt of Beethoven's Symphony No. 5 in C minor, Mvt I. Silence and timing are used to make the music breathe. See Supplementary Video 4 in the Supplementary Materials to hear the music presented in this Figure.

**Pauses** occur analogously in speech and music as segmentation devices that add space between two adjacent structures. In music, this concept is related to the timing of the notes in a piece and is executed by the performer *via* lingering on notes or by using silence. Silence is a very powerful tool in music. It is used for separating musical elements, to hold the audience in suspense, or for other expressive effect. Pause are executed before, during, or after a musical event. When notated in the music, pauses are indicated by a comma or a *fermata* symbol placed above a note or a rest. The term *breath* is used either figuratively or literally in relation to the performer's breath when executing a musical passage (Figure 6). The duration of a pause is sometimes specified by the composer but the performer makes the ultimate decision of how long it should be depending on the unique circumstances of a performance.

Transitions and pauses may be marked with the *Region* annotation type in CosmoNote. Participants are able to mark the beginnings and ends of these structures and label them accordingly. When the starting/ending times are not clear, especially for transitions, it is recommended to place them at the outermost value possible. Overlapping regions are allowed as needed.

### 3.2. Annotating Prominence

Prominence generally characterizes an emphasis drawn toward a certain element of a whole. Prominence in speech is important because speakers are capable of changing the meaning of an utterance by assigning more weight to a certain word or by changing their intonation. In music, experienced performers may make musical structures stand out in a way that helps to resolve ambiguities, particularly in musical meter (Sloboda, 1985). They may also introduce focal points by assigning more weight to a note or a chord.

For this description, we divide musical prominence into two sub-categories: vertical or horizontal based on their temporality. Events that may or may not segment the music but that are easily recognized as belonging to a single moment, using timing and dynamics, are classified as vertical prominence. On the other hand, attention drawn to a particular sequential structure such as a melodic line that cannot be pinpointed to a clear moment, is categorized as horizontal prominence. Since prominence can be viewed as either vertical or horizontal, all vertically prominent structures may be marked using *Regions* (if a preparatory stage exists) and *Boundaries (or Comments)* while horizontally emphasized ones may be marked with the *Note group* annotation type (e.g., notes in an important motive or a salient melody).

As was the case with segmentation (see Section 3.1), we will concentrate on the point of view of the performer in the following descriptions of common prominence creation techniques (stress, melodic salience, and tipping points) that listeners are likely to recognize while annotating prominence.

**Figure 7:**
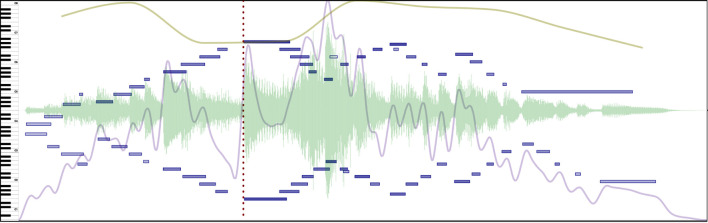
Stress is marked by a comment (red dotted line) at the pinnacle of the progression in this excerpt of Beethoven's Variation No. III from 32 Variations in C minor. See Supplementary Video 5 in the Supplementary Materials to hear the music presented in this Figure.

**Stress** is an emphasis on a particular element to make it more prominent than those around it. In speech, stress is used to help parse words in a language like English where syllables and words can be stressed to alter the meaning of an utterance (Ashby, 2011). In music, this category is close to how Drake and Palmer (1993) define *rhythmic grouping*, which focuses on event intensity/duration and *metric accents*, which focus on higher order regularities in a sequence. Thus, stress may be indicated by a combination of performer actions like an increase in sound intensity, duration, or even a change in timbre, as seen in Figure 7.

**Figure 8:**
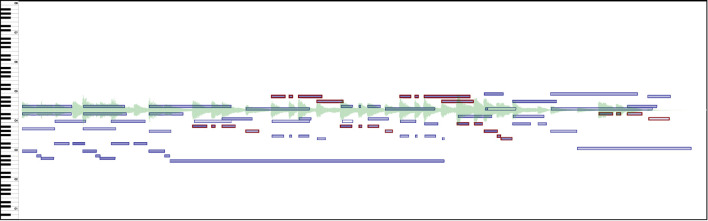
Melodic salience highlighted in note groups (light red rectangles) as the notes of the main motive are made more present by the performer on an excerpt of Chopin's Ballade No. 2 in F major. See Supplementary Video 6 in the Supplementary Materials to hear the music presented in this Figure.

**Melodic salience** is a special case of prominence dedicated to the melody of a piece. It relates to the concept of *melodic accents* by Drake and Palmer (1993). Melodic salience, as shown in Figure 8, may be recognized by an increase in loudness and duration (the notes of the main voice in a melody are usually louder and longer) or a variation in timbre (by using a different touch/technique or a different instrument altogether). Performers can indicate melodic salience in piano performances by systematic variation of intensity and duration, even within hands, to enhance the melody (Repp, 1996).

**Figure 9:**
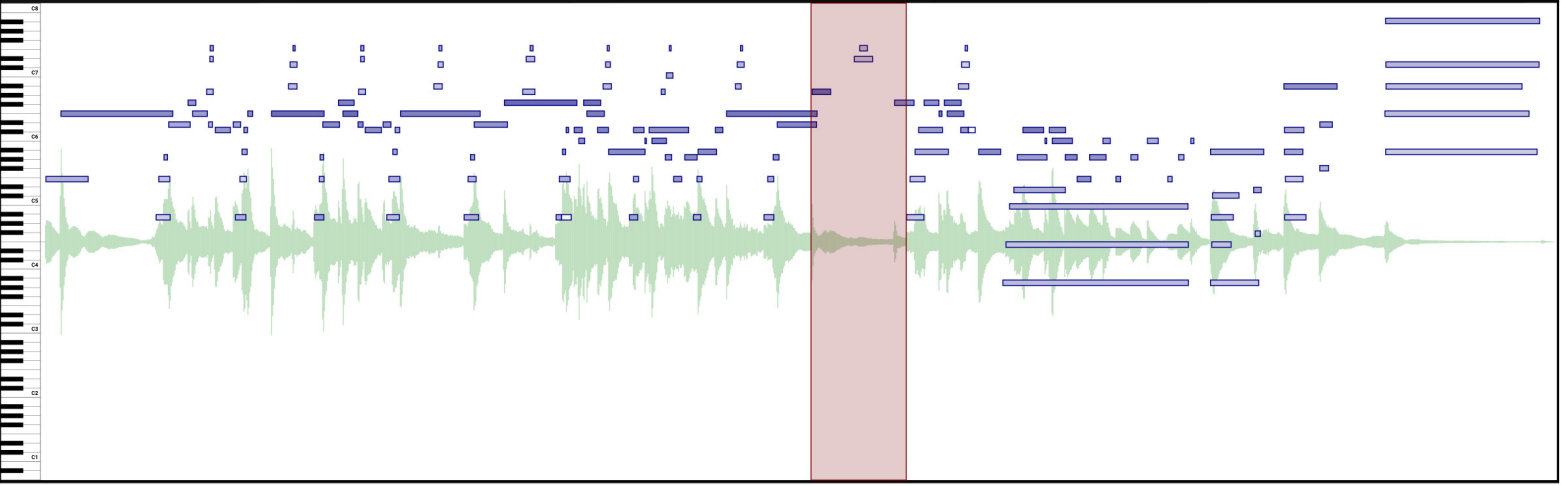
A tipping point marked with a region (light red rectangle) on Grieg's Solveig's Song evidences how a musical moment is stretched to its limit. See Supplementary Video 7 in the Supplementary Materials to hear the music presented in this Figure.

**Tipping points**, as defined by Chew (2016), are cases of “extreme pulse elasticity” where musical time is suspended in an unstable state beyond which a return to the pulse is inevitable. As such, they are frequently present in musical transitions (a clear distinction exists before and after the tipping point) and musical pauses (a tipping point created at the moment a pause can no longer be stretched). Figure 9 shows a passage with a melodic tipping point, where the performer plays with the listeners' expectations and delays closure of the sub-phrase.

### 3.3. Potential Annotation Strategies

Participants are presented with annotation instructions to direct them in doing the annotation task. Yet the specifics of their annotation placement are driven by their individual strategies. The following is an excerpt of the general annotation instructions that participants read when they enter the main annotation campaign in CosmoNote: “*Please mark the boundaries that you hear in the music, and indicate the strength of each boundary. You may be presented with information layers such as the notes, tempo or loudness, but your ear should be your main guide*”. Although there are numerous ways to reach this goal, we will center on two branching strategies that are common to all annotations, namely real-time vs. retrospective annotations and analytic vs. intuitive mindsets. These methods will be examined using actual experiences that CosmoNote participants (software testers) had during the development phase, which are used to anticipate and deal with problems that future annotators may confront.

Sometimes, participants wanted to listen to the whole piece, from start to finish, before placing any annotation. These participants found it easier to first concentrate their full attention on the music, forming their own mental model of the piece's structure and of what the performer meant to communicate, and only then proceeding to the annotation stage. This retrospective annotation process could take longer but listeners who adopted it would have to revise their work less. In contrast, some participants preferred annotating in real-time, which meant marking structures while listening to the audio, even for the first time. Since music was listened to and understood retroactively, if listeners were not already familiar with the music, they would need to go back again to correct their work after becoming more familiar with it. To maximize the benefits of real-time and retrospective annotations, we recommend marking only the biggest boundaries on the first play-through. Once the larger segments are defined, annotators may go back, focus on smaller segments and repeat the process, correcting mistakes as needed.

There is also the question of whether to analyze the music intellectually or to annotate by intuition. An analytical approach will vary depending on the person's musical knowledge, experience or formal training. It is important to note that annotating analytically does not mean using traditional music theory or score structure analysis, nor is it about finding repeating patterns within the sounds. Annotating analytically means thinking deeply about the performer-made segments and prominent structures in the music. The counterpart of this strategy is a more intuitive, spontaneous annotation, where citizen scientists try to reduce their cognitive load, be more comfortable, focus less on being *right* and embrace the subjectivity of the task. The potential drawback of this strategy is that listeners could end up annotating the emotions conveyed by the music, which is not the purpose of this methodology. We recommend some balance between the two, where neither attentive listening nor spontaneity are privileged one over the other.

Since music is primarily an auditory stimulus, participants are discouraged from placing annotations only by relying on visual cues. However, no matter the approach, any complementary information (e.g., visuals or cues like boundary sounds) should be understood more as advice than a prescribed way to perform the task since we do not wish to impose a fixed way in which annotators use the tools in CosmoNote. In fact, we want them to explore the possible uses of the tools and give us feedback on how they use them. Ultimately, any instructions or strategy should be mostly used to help externalize the intuitions that are formed while listening to the music. This is why it is made clear, at various stages, that the most important aspect of the musical annotation process for participants is to trust their ears.

## 4. Anticipated Results

### 4.1. Data Structure and Analysis

**Figure 10:**
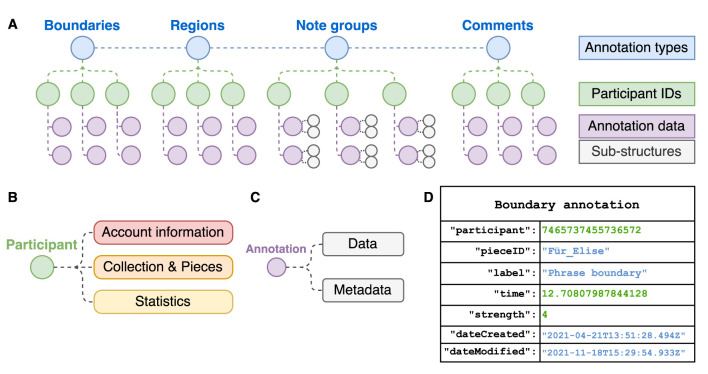
Data structure for the CosmoNote Annotations as organized in json objects. **(A)** A representation of nested data in each of the four annotation types; **(B)** A participant object contain information about its Account, Collection & Pieces, and aggregated Statistics; **(C)** Annotation objects contain data (e.g., timestamps, labels) and metadata (e.g., date of creation and last modification); **(D)** An example boundary annotation json object.

Data from annotations collected in CosmoNote is organized in a secure database and exported using a json (JavaScript Object Notation) format for its analysis. Structures of json arrays of paired name/value objects ensure great flexibility and modularity. Data for each of the four annotation types is classified by pseudonymized participant ID numbers. Objects contain primary properties (data) like timestamps and labels, and secondary properties (metadata) such as creation and modification dates. For example, note group annotations not only contain an onset time but also sub-structures like the MIDI note identifier for each note in a group. A scheme of the annotation data structures is shown in Figure 10. More properties may be incorporated as CosmoNote evolves, is adopted by more users, or as the need arises. For instance, the data structure does not currently contain information on which music features the annotator chose to display and when. However, since the ability to track user interaction involves a non-trivial amount of development work, it will be part of future work.

For each piece, our main variables are distributed over the quantity and location (time, and pitch when applicable) of each data point from the four types of annotations, each related to a participant and containing auxiliary properties (as described above). In addition, the music features that are computed for visualizations in CosmoNote (MIDI data, loudness, tempo, and harmonic tension curves) constitute important synchronized time-based information for the analysis of the annotation data. To establish relevant relationships between aggregated annotation data, high-level prosodic features, and low-level musical features and acoustic properties, we will use techniques such as change point analysis, cluster analysis, and multiple regressions.

### 4.2. Accuracy and Precision of Annotations

This protocol relies on manual annotations of musical prosody. This approach is the most appropriate to explore questions about intuitions humans have that machines do not (i.e., the recognition of subtle cues of prominence in ambiguous musical structures). This means that accuracy is not strictly a problem for this method since we do not assume there is a right answer for the placement of annotations. However, precision among the annotations is an issue since the precise timing of segmentation or prominence annotations could differ across participants.

Imprecision will be accounted for by creating a large profile of annotations for a single piece. However, because of the possible deviations in timestamps, each annotation will be weighted to create this profile, where a tolerance proxy is set. For example, ±3 s or the equivalent of 1 bar of a piece with quadruple meter at 80 bpm (Ong, 2006, p. 58). Comparing the mean profile with each individual profile will help detect outliers in an individual profile. The data will also be checked for internal consistency.

### 4.3. A Library of Examples

Another outcome of the citizen science studies is the creation of a performance-oriented library of musical prosody examples. It is an easily accessible, free resource intended for music professionals (performers, composers, musicologists, music educators), music enthusiasts (without formal musical training), and the public at large. This nascent library is composed of four main parts: The raw MIDI and audio data obtained from the pieces in the datasets described in Section 2.1, the computed musical features (loudness, tempo, harmonic tension) as described in Section 2.2.1, individual annotations collected using the protocol detailed throughout Section 3, and aggregated data derived from the analysis of individual annotations, as specified in Section 4.2.

Anyone who accesses this library will be able to listen to the music and see the annotations as they were created. They will have total control over what representation they see, how to layer multiple visualizations, which type of annotation is shown, and which labels were assigned at a given point. Users will also be able to use zoom controls to interact with the content at their preferred time scale, and see the aggregation of multiple users' annotations for the same piece when they are available in CosmoNote. The library already contains more than a dozen examples of musical structures and can be accessed through a playlist in YouTube.^4^

### 4.4. New Models and Conventions for Musical Prosody

We believe that large scale usage and adoption of our method presents a step toward improving current practices for thinking about and annotating musical prosody in performance. Using the citizen science paradigm, we hope to build a community around the research of these structures in performed music. Each and every person that participates in our studies may come with their own motivations (e.g., passing time, learning about music, listening to their favorite artist) but they will also contribute to the same shared goal of understanding musical prosody in performed music. In the process of annotating the music, citizen scientists will refine our protocol and facilitate its wide adoption as part of a set of conventions to annotate musical prosody.

The data generated from our method will be used to inform new models for prosody perception and production both from theoretical (rule-based) and data-driven (machine learning) analyses. Theoretical models will be informed by our results, representing abstractions in performance that use musical prosody, and that are difficult for machines to recognize. Our analysis will extract a distribution of prosodic structures from which observed patterns can be described and modeled *via* algorithms that can be validated by expert annotators. On the other hand, data-driven models will be constructed by using human annotations to train machines to more naturally identify musical prosody. In any case, our objective is to use our models to mediate the relationship between prosodic structures and acoustic variations from a different perspective, that helps composers, performers, and music lovers in general, to enhance their understanding of the role of prosody in music performance.

## 5. Discussion

In this article, we have presented a novel and scalable method to represent, identify, annotate and analyze musical prosody in performed music from a top-down, human-centered perspective. This method is supported by an innovative web-based platform that combines traditional and current music representations to improve the annotation experience. The results of data collection studies using this method will contribute to better understanding of how humans perceive prosodic structures in performed music. We aim to construct models that explain how these structures are created and used by performers in their real-world practice. The data we collect will also be used for the development of a library of musical prosody examples that will form the basis for formulating a musical prosody annotation convention.

Many of the advantages of our method are related to how we collect the data. Since the process is self-paced, annotators are not constrained by a fixed time limit. The instructions explain what musical prosody is and provide concrete examples of how prosody may be presented in expressive performance. Thus, annotators can afford to listen attentively, think about their choices, and even modify their annotations during a subsequent markup session. The music visualizations and training examples provide cognitive scaffolding (Yelland and Masters, 2007) for users with the various visual layers giving complementary information. For example, the piano roll visualization layer is helpful to single out the note onsets and intensities of a prominent melody but only gives vague information regarding tempo while more specific information about tempo is provided by the tempo layer. It is worth noting that piano music was chosen for the simplicity of aligning event-based (MIDI) information with an audio signal. However, any music audio can be depicted on the interface and the tools work equally well for denoting expressiveness in music for other instruments, including voice. The main obstacle in this case would be the difficulty of deriving precise note onset and offset information aligned with the music audio, but that is not insurmountable (e.g., with manual annotation). We anticipate that the ways in which the tool is used could evolve to encapsulate many more expressive devices appropriate for other instruments, where the ideas of segmentation and prominence still apply. With regard to the current interface, the tools available in CosmoNote are easy to use by virtue of their simplicity. The audio controls are similar to any music reproduction software and annotations are placed with a click of the mouse or a keyboard shortcut.

The challenges we encounter are typical of many citizen science projects and include ensuring data quality and sustaining community engagement. As for the data itself, the types of structures that can be marked are potentially limited by technical constraints, the difficulty, and duration of the task. For example, even though there is no time-limit, if a specific action in CosmoNote is cumbersome, it can be perceived as tiring or frustrating. Solutions for these problems are generally complex; one approach is to address them methodically and iteratively at the software development level. For example, if we find that one prosodic marker/annotation type is being used rarely, we could first investigate why by getting direct feedback from the feedback questionnaires and then make changes accordingly. If annotators do not find an annotation type/visualization useful for marking musical prosody, since the representation captured by this feature is not being correctly communicated or is redundant, the feature can be redesigned or removed. The same process would apply if a given interaction is not consistent/intuitive and the user experience could be improved by simplifying the interaction or introducing annotation types in a progressive manner in order to reduce frustration or fatigue.

Our annotation protocol, the training content, and the feedback surveys are means to bolster data validity and reliability (Balázs et al., 2021). For instance, the first campaign was launched to gather annotations while serving the complementary purpose of providing information for improving people's interactions with CosmoNote, thus encouraging further contributions. We are in the process of building a community that shares our goals of understanding how music structures are created and shaped in performance through musical prosody. We rely on members' contributions and feedback; it is with the cooperation of both expert and non-expert annotators that we will iterate and improve upon the components of our annotation method. To ensure sustained community engagement, more collaborative features that connect citizen scientists are planned for the future versions of CosmoNote.

Our method is an ambitious attempt at discerning abstract musical structures in performed music through listeners' annotations. CosmoNote is a flexible and extensible tool suitable for representing and annotating musical prosody in real-world performances. By using it to explore how humans apprehend segmentation and prominence introduced in performance, we will have the means to design models that capture the complex relationships of these structures with musical features and their acoustic properties in a comprehensive way. Subsequent phases in our studies will build iteratively upon the results of previous ones, ensuring continued progress of the annotation method. The long term goal of this research is to open new paths for the general public to think about what is being communicated in expressive music through the performer's segmentation of musical ideas and creation of musical prominence, and to offer new ways to explore and talk about performed music in general.

## Data Availability Statement

The Musical questionnaire and Feedback questionnaire, as well as videos showing playback of the musical examples presented in Figures 1, 4–9 can be found in the Supplementary Material. Further inquiries can be directed to the corresponding author.

## Author Contributions

LF developed CosmoNote. DB and EC created the annotation protocol and annotated the pieces. EC played the examples. DB recorded them. All authors contributed to the writing and to the design of CosmoNote. All authors contributed to the article and approved the submitted version.

## Funding

This result was part of the project COSMOS that has received funding from the European Research Council under the European Union's Horizon 2020 research and innovation program (Grant Agreement No. 788960).

## Conflict of Interest

The authors declare that the research was conducted in the absence of any commercial or financial relationships that could be construed as a potential conflict of interest.

## Publisher's Note

All claims expressed in this article are solely those of the authors and do not necessarily represent those of their affiliated organizations, or those of the publisher, the editors and the reviewers. Any product that may be evaluated in this article, or claim that may be made by its manufacturer, is not guaranteed or endorsed by the publisher.

## Footnotes

^1^ https://cosmonote.ircam.fr

^2^ https://cosmonote.ircam.fr/agreement.html

^3^ https://www.youtube.com/channel/UCEOzV1aQ4zOM2RLUq-AV0Og

^4^ https://youtube.com/playlist?list=PLR0hNEZTO56Nbjc02ciW_EPxrWnmnKlp1

## Supplementary Material

Supplementary Video 1 CosmoNote's interface: Video corresponding to Figure 1: The main CosmoNote interface showing an annotated excerpt of Beethoven's Für Elise. Different visualizations and annotation types are displayed.

Supplementary Video 2 Boundaries: Video corresponding to Figure 4: Excerpt of Beethoven's Symphony No. 5 in C minor, Mvt II. Boundaries (red vertical lines) in this example are assigned according to the strength of the change. Stronger boundaries have a thicker, less opaque red color.

Supplementary Video 3 Transition: Video corresponding to Figure 5: Excerpt of a long transition marked with a region (light red rectangle) between Movements III and IV of Beethoven's Symphony No. 5 in C minor.

Supplementary Video 4 Pauses: Video corresponding to Figure 6: Two pauses marked with regions (light red rectangles) in an excerpt of Beethoven's Symphony No. 5 in C minor, Mvt I. Silence and timing are used to make the music breathe.

Supplementary Video 5 Stress: Video corresponding to Figure 7: Stress is marked by a comment (red dotted line) at the pinnacle of the progression in this excerpt of Beethoven's Variation No. III from 32 Variations in C minor.

Supplementary Video 6 Melodic salience: Video corresponding to Figure 8: Melodic salience highlighted in note groups (light red rectangles) as the notes of the main motive are made more present by the performer on an excerpt of Chopin's Ballade No. 2 in F major.

Supplementary Video 7 Tipping point: Video corresponding to Figure 9: A tipping point marked with a region (light red rectangle) on Grieg's Solveig's Song evidences how a musical moment is stretched to its limit.

Supplementary Datasheet 1 Musical Questionnaire: Example of CosmoNote's Musical Questionnaire, adapted from the Goldsmiths Musical Sophistication Index (Gold-MSI, by Müllensiefen et al., 2014).

Supplementary Datasheet 2 Feedback Questionnaire: Example of CosmoNote's Feedback Questionnaire for a boundary annotation task.

## References

[B1] Ashby P. (2011). Beyond the segment, in Understanding Phonetics, eds Comrie B. Corbett G. (London: Routledge), 159–163.

[B2] Balázs B. Mooney P. Nováková E. Bastin L. Jokar Arsanjani J. (2021). Data quality in citizen science, in The Science of Citizen Science, eds Vohland K. Land-Zandstra A. Ceccaroni L. Lemmens R. Perello J. Ponti M. Samson R. Wagenknecht K. (Cham: Springer), 139–157. 10.1007/978-3-030-58278-4_8

[B3] Beckman M. E. Ayers G. (1997). Guidelines for ToBI labelling. OSU Res. Found. 3, 30.

[B4] Boersma P. Van Heuven V. (2001). Speak and unspeak with praat. Glot Int. 5, 341–347.

[B5] Brugman H. Russel A. Nijmegen X. (2004). Annotating multimedia/multi-modal resources with Elan, in LREC (Lisbon), 2065–2068.

[B6] Cambouropoulos E. (2006). Musical parallelism and melodic segmentation: a computational approach. Music Percept. 23, 249–268. 10.1525/mp.2006.23.3.249

[B7] Cannam C. Landone C. Sandler M. (2010). Sonic visualiser: an open source application for viewing, analysing, and annotating music audio files, in Proceedings of the 18th ACM International Conference on Multimedia (New York, NY: ACM), 1467–1468. 10.1145/1873951.1874248

[B8] Cartwright M. Pardo B. Mysore G. J. Hoffman M. (2016). Fast and easy crowdsourced perceptual audio evaluation, in 2016 IEEE International Conference on Acoustics, Speech and Signal Processing (ICASSP) (Shanghai: IEEE), 619–623. 10.1109/ICASSP.2016.7471749

[B9] Chew E. (2016). Playing with the edge: tipping points and the role of tonality. Music Percept. 33, 344–366. 10.1525/mp.2016.33.3.344

[B10] Couprie P. (2012). Eanalysis: aide á l'analyse de la musique électroacoustique, in Journées d'Informatique Musicale (Mon), 183–189.

[B11] Drake C. Palmer C. (1993). Accent structures in music performance. Music Percept. 10, 343–378. 10.2307/40285574

[B12] Fillon T. Simonnot J. Mifune M.-F. Khoury S. Pellerin G. Le Coz M. (2014). Telemeta: an open-source web framework for ethnomusicological audio archives management and automatic analysis, in Proceedings of the 1st International Workshop on Digital Libraries for Musicology (New York, NY), 1–8. 10.1145/2660168.2660169

[B13] Fyfe L. Bedoya D. Guichaoua C. Chew E. (2021). CosmoNote: a web-based citizen science tool for annotating music performances, in Proceedings of the International Web Audio Conference, WAC '21, eds Joglar-Ongay L. Serra X. Font F. Tovstogan P. Stolfi A. Correya A. Ramires A. Bogdanov D. Faraldo A. Favory X. (Barcelona: UPF), 1–6.

[B14] Haklay M. M. Dörler D. Heigl F. Manzoni M. Hecker S. Vohland K. (2021). What is citizen science? The challenges of definition, in The Science of Citizen Science, eds Sui D. Elwood S. Goodchild M. (Dordrecht: Springer), 13–33. 10.1007/978-3-030-58278-4_2

[B15] Herremans D. Chew E. (2016). Tension ribbons: quantifying and visualising tonal tension, in Second International Conference on Technologies for Music Notation and Representation (TENOR) (Cambridge: TENOR), 10.

[B16] Kennedy M. Kennedy J. (2013). The Oxford Dictionary of Music. Oxford: Oxford University Press. 10.1093/acref/9780199578108.001.0001

[B17] Lerdahl F. Jackendoff R. (1996). A Generative Theory of Tonal Music. Cambridge, MA: MIT Press. 10.7551/mitpress/12513.001.0001

[B18] Migliore O. Obin N. (2018). At the interface of speech and music: a study of prosody and musical prosody in rap music, in Speech Prosody, eds Klessa K. Bachan J. Wagneer A. Karpiski M. Sledzinski D. (Poznan: International Speech Communication Association), 557–561. 10.21437/SpeechProsody.2018-113

[B19] Müllensiefen D. Gingras B. Musil J. Stewart L. (2014). The musicality of non-musicians: an index for assessing musical sophistication in the general population. PLoS ONE 9, e89642. 10.1371/journal.pone.0089642 24586929 PMC3935919

[B20] Nakamura E. Yoshii K. Katayose H. (2017). Performance error detection and post-processing for fast and accurate symbolic music alignment, in ISMIR (Suzhou), 347–353.

[B21] Ong B. S. (2006). Structural Analysis and Segmentation of Music Signals. Barcelona: Citeseer.

[B22] Palmer C. Hutchins S. (2006). What is musical prosody? in Psychology of Learning and Motivation, Vol. 46, ed Ross B. H. (Urbana, IL: Academic Press), 245–278. 10.1016/S0079-7421(06)46007-2

[B23] Pampalk E. (2004). A Matlab toolbox to compute music similarity from audio, in ISMIR (Barcelona), 254–257.

[B24] Repp B. H. (1996). Patterns of note onset asynchronies in expressive piano performance. J. Acoust. Soc. Am. 100, 3917–3932. 10.1121/1.417245 8969489

[B25] Rohrmeier M. Pearce M. (2018). Musical syntax I: theoretical perspectives, in Springer Handbook of Systematic Musicology, ed Bader R. (Heidelberg: Springer), 473–486. 10.1007/978-3-662-55004-5_25

[B26] Senabre Hidalgo E. Perelló J. Becker F. Bonhoure I. Legris M. Cigarini A. (2021). Participation and co-creation in citizen science, in The Science of Citizen Science, eds Vohland K. Land-Zandstra A. Ceccaroni L. Lemmens R. Perello J. Ponti M. Samson R. Wagenknecht K. (Cham: Springer), 199–218. 10.1007/978-3-030-58278-4_11

[B27] Sloboda J. A. (1985). Expressive skill in two pianists: metrical communication in real and simulated performances. Can. J. Psychol. 39, 273. 10.1037/h0080062

[B28] Speer S. Blodgett A. (2006). Prosody, in Handbook of Psycholinguistic, eds Traxler M. J. Gernsbacher M. A. (London: Elsevier), 505–537. 10.1016/B978-012369374-7/50014-6

[B29] Spencer P. Temko P. M. (1994). A Practical Approach to the Study of Form in Music. Long Grove, IL: Waveland Press.

[B30] Wang C.-I. Mysore G. J. Dubnov S. (2017). Re-visiting the music segmentation problem with crowdsourcing, in ISMIR (Suzhou), 738–744.

[B31] Yelland N. Masters J. (2007). Rethinking scaffolding in the information age. Comput. Educ. 48, 362–382. 10.1016/j.compedu.2005.01.010

