# Supplementary material for: A Perceiver-Centered Approach for Representing and Annotating Prosodic Functions in Performed Music
Source: Front Psychol. 2022 Jul 21;13:886570. doi: 10.3389/fpsyg.2022.886570 (PMC9355529; doi:10.3389/fpsyg.2022.886570)
Supplement: Supplementary Datasheet 1 — Musical Questionnaire: Example of CosmoNote's Musical Questionnaire, adapted from the Goldsmiths Musical Sophistication Index (Gold-MSI, by Müllensiefen et al., 2014). [file Data_Sheet_1.PDF]

CosmoNote

Musical Questionnaire

The purpose of this short self-report questionnaire is:
a) to quantify your amount of musical engagement and behaviour in its many possible facets
b) to record your self-assessed level amongst various musical skills.

Please answer these questions by selecting the most appropriate option for you.

Agreement questions

Question	Completely Disagree<br>1	Strongly Disagree<br>2	Disagree<br>3	Neither Agree nor Disagree<br>4	Agree<br>5	Strongly Agree<br>6	Completely Agree<br>7
I spend a lot of my free time doing music related activities	○	○	○	○	○	○	○
I can reproduce (e.g., sing, whistle, hum) music from memory	○	○	○	○	○	○	○
I am able to hit the right notes when I reproduce a melody (e.g., sing, whistle, hum) along with a recording	○	○	○	○	○	○	○
I can compare and discuss differences between two performances or versions of the same piece of music	○	○	○	○	○	○	○
I am not able to reproduce a melody (e.g., sing, whistle, hum) in harmony when somebody is singing a familiar tune	○	○	○	○	○	○	○

Question	Completely Disagree<br>1	Strongly Disagree<br>2	Disagree<br>3	Neither Agree nor Disagree<br>4	Agree<br>5	Strongly Agree<br>6	Completely Agree<br>7
I am able to identify what is special about a given musical piece	○	○	○	○	○	○	○
When I reproduce a melody (e.g., sing, whistle, hum), I have no idea whether I'm in tune or not	○	○	○	○	○	○	○
Music is kind of an addiction for me - I couldn't live without it	○	○	○	○	○	○	○
After hearing a new song two or three times, I can usually reproduce its melody (e.g., sing, whistle, hum) it by myself	○	○	○	○	○	○	○

Multiple choice

I engaged in regular, daily practice of a musical instrument (including voice) for _____ years

At the peak of my interest, I practised _____ hours on my primary instrument (including voice)

I have had formal training in music theory for _____ years

I can play the following number of musical instruments (including voice)

Demographic data

What is your current occupational status

○ Still at School

- At University
- In Full-time employment
- In Part-time employment
- Self-employed
- Homemaker/full time parent
- Unemployed
- Retired

What is the musical genre you mainly listen to?

- Rock/Pop
- Jazz
- Classical Music
- Hip-Hop/Rap
- Electronica
- Other

What is the Highest educational qualification you have attained?

- Did not complete any school qualification
- Completed first school qualification (e.g. GSCE/Junior High School)
- Completed second school qualification (e.g. A levels / High School
- Undergraduate degree of professional qualification
- Postgraduate degree
- I am still in education

Please select your age group

Please select your gender

Please select your current country of residence

Finish
