# Supplementary material for: A Perceiver-Centered Approach for Representing and Annotating Prosodic Functions in Performed Music
Source: Front Psychol. 2022 Jul 21;13:886570. doi: 10.3389/fpsyg.2022.886570 (PMC9355529; doi:10.3389/fpsyg.2022.886570)
Supplement: Supplementary Datasheet 2 — Feedback Questionnaire: Example of CosmoNote's Feedback Questionnaire for a boundary annotation task. [file Data_Sheet_2.PDF]

CosmoNote

Feedback Questionnaire

(for a boundary annotation task)

Thank you for having finished annotating this collection!

Please take a minute to answer the following questions by selecting the most appropriate option for you.
This will help us to better understand your results.

User Experience

We would like to know how was your overall experience using CosmoNote.
Using a scale from 1 (completely disagree) to 7 (completely agree) answer the following questions:

**Question**	**Completely Disagree**<br>**1**	**Strongly Disagree**<br>**2**	**Disagree**<br>**3**	**Neither Agree nor Disagree**<br>**4**	**Agree**<br>**5**	**Strongly Agree**<br>**6**	**Completely Agree**<br>**7**
Annotating music in CosmoNote was enjoyable	○	○	○	○	○	○	○
Recognizing and marking boundaries was easy	○	○	○	○	○	○	○
CosmoNote was easy to use	○	○	○	○	○	○	○
The music's sound quality was good	○	○	○	○	○	○	○

Please elaborate on your experience using our platform *(optional)*

Suggestions for improving the CosmoNote User Experience *(optional)*

Annotation Strategies

We are also interested in how you approached the annotation task(s).

What were your main strategies for marking boundaries?

Some visualization options are on/off by default, but you can also toggle between them.

We would like you to rate how much you used the available visualizations to actually inform your annotations.
Using a scale from 1 (never used it) to 5 (always used it) answer the following questions:
(if the visualization wasn't shown, then use N/A)

**Question**	**Never 1**	**Rarely 2**	**Sometimes 3**	**Often 4**	**Always 5**	**N/A**
Audio waveform (pale-green)	○	○	○	○	○	○
Piano roll (notes - blue rectangles)	○	○	○	○	○	○

Question	Never<br>1	Rarely<br>2	Sometimes<br>3	Often<br>4	Always<br>5	N/A
Pedals (pale areas)	○	○	○	○	○	○
Loudness (purple curve)	○	○	○	○	○	○
Tempo (olive curve)	○	○	○	○	○	○
Tension	○	○	○	○	○	○

Please elaborate on your choices e.g., why you preferred a visualization over another *(optional)*

Familiarity and Preference

We are interested in knowing if you had heard this music before and how much you enjoyed it.
Using a scale from 1 (completely disagree) to 7 (completely agree) answer the following questions:

Question	Completely Disagree<br>1	Strongly Disagree<br>2	Disagree<br>3	Neither Agree nor Disagree<br>4	Agree<br>5	Strongly Agree<br>6	Completely Agree<br>7
The music was new to me	○	○	○	○	○	○	○
I like the music I annotated	○	○	○	○	○	○	○

Please elaborate on your previous knowledge of the music you just annotated *(optional)*

Further Comments

Tell us if you have any additional comments about your experience annotating this collection *(optional)*

Submit / Reset

We hope to see you again soon!
